# Model of Social Support for Patients Treated for Cancer

**DOI:** 10.3390/cancers13194786

**Published:** 2021-09-24

**Authors:** Małgorzata Pasek, Lilia Suchocka, Krzysztof Gąsior

**Affiliations:** 1 Department of Nursing, Faculty of Health, University of Applied Sciences in Tarnów, 33-100 Tarnów, Poland; 2 Faculty of Education and Psychology, Jan Kochanowski University of Kielce, 25-029 Kielce, Poland; liliasuchocka@ibnps.eu (L.S.); gasior@psychoterapia24.com (K.G.)

**Keywords:** social support, cancer, oncological treatment, theoretical model, structural equations

## Abstract

**Simple Summary:**

The experience of being diagnosed with a neoplastic disease and the associated cancer treatment, which is worrying and tough for the patient and their family, causes many difficult situations. Studying the multidimensional approach to social support in oncological patients has allowed for the development of a research model of social support using structural equations. Social support is an important variable in coping with cancer.

**Abstract:**

Social support can be one of the main factors in better assessing the quality of life at any stage of the recovery process. It should meet the patient’s needs so that they can develop constructive methods of coping with the disease. In order to explain the factors influencing social support for cancer patients, a theoretical research model was formulated. It is presented in a graphic form in this article. In order to verify the model, the authors’ questionnaire and other standardised questionnaires were used. The experimental group consisted of 170 hospitalised oncological patients being treated for cancer. Personality structure, through its relationship with state of health (0.40) and attitude developed to the disease (0.64), influenced the support experienced by the treated patients (0.40). The surveyed patients, who were characterised by positive self-esteem and experience of life satisfaction despite various difficult situations, perceived to a greater degree the emotional and instrumental support provided to them. During cancer treatment, those patients who showed a need for help and did not experience negative emotions were characterised by an increased need for support. The research results can be introduced into evidence-based medical practice, which could significantly improve the quality of nursing and medical care for patients.

## 1. Introduction

Neoplastic diseases are among the leading causes of death in the world [1]. Thanks to the introduction of multidisciplinary methods of treatment, their status has changed, and they have become chronic diseases. This situation has significantly contributed not only to the extension of patients’ lives but also to the emergence of new problems for patients and their relatives.

The experience of being diagnosed with cancer and the associated treatment, which is worrying and difficult for the patients and their families, prompted the authors to search for the factors influencing the creation of a healing path, in which internal and external resources would be consciously used to deal with a long-term stressful situation. One such factor is social support, which for the purposes of this work is defined in terms of perception. Defining perception in this way makes it possible to take into account the important aspects of this concept: the level of support obtained from various sources and the degree of an individual’s satisfaction with this support, which is particularly beneficial in research on the buffering effect of stress on mental health [2].

According to social support theory, social support should be meet patients’ needs to allow them to develop optimal methods of coping with a disease. Excessive support in a human’s life may have a negative impact on their activity and may cause a loss of independence [3]. Research shows that the optimisation of support allows patients to develop a healthy approach to treatment and recovery. Social support becomes a moderator and has a positive effect on psychological functioning even after the occurrence of a stressor [4].

Social support can be one of the main factors in better assessing the quality of life at any stage of the recovery process [5]. Research by Pehlivan et al. has shown that patients with a high level of perceived social support did not feel hopeless or lonely [6]. The absence of these conditions is important in coping with cancer.

Research indicates that receiving support from family and friends including calmness, comfort, and problem-solving in the post-diagnosis period is an important resource that can help cancer survivors find positive meaning in their cancer experience [7]. Social support was also mentioned as a factor that significantly contributed to disease management and treatment in women with breast cancer. The support available from family members, friends, neighbours, and health professionals was essential. Social support is an important determinant of good care. When traumatic treatment was necessary, social support increased its effectiveness by helping patients gain a positive view of their health and cope better with cancer. The necessity of adapting to the individual needs of patients is also emphasized [8].

A low level of perceived social support in cancer patients resulted in a significantly higher level of depression, lower scores on all functional scales, higher scores on all symptom scales, and lower scores on the overall health and quality of life scale. Perceived social support reduced the adverse psychological effects of treatment [9,10].

In their research, Halkett et al. have stated that patients diagnosed with a brain tumour may have unique needs. These relate, among others, to the scope of the support, which may change over time [11], indicating that the level and type of support should be monitored.

Another study has identified some predictors for assessing psychosocial adjustment and care for patients with physical illnesses. Conclusions for daily clinical practice include paying particular attention to psychosocial support for patients with physical illnesses, people with negative perceptions of their illnesses, and those suffering from depression [12,13].

In the cited study, cancer patients expressed their expectations. These concerned the need for companionship (45%), empathy (33%), support in the field of home care (28%), information support (16%), the same treatment (15%), and assistance during doctor’s visits (13%) [14].

Among the sources of social support, the surveyed patients indicated healthcare professionals, which made it possible to demonstrate a correlation between the support received from them, the need for and satisfaction with the support received, and all important aspects of the quality of life [15].

The available scientific reports examining the multidimensional aspect of social support indicate the need to update the research, even if it has indicated that social support is assessed as satisfactory. The aim of such research is to maintain the wellbeing of patients [16].

The research results can be introduced into evidence-based medical practice, which could significantly improve the quality of nursing and medical care for patients.

The aim of our study was to investigate the factors influencing the multidimensional aspects of social support for patients hospitalised in oncology wards and undergoing cancer treatment.

## 2. Materials and Methods

### 2.1. Hypotheses

In connection with the aim of the study, the following research hypotheses were formulated:

**Hypothesis** **1** **(H1).** *The determinants of perceived available support consisting of perceived emotional and instrumental support are positive orientation and the absence of negative emotional states in the treatment process*.

**Hypothesis** **2** **(H2).** *The respondents’ need and search for support are influenced by a general need for help, a lack of extreme negative emotions (such as anger), and openness in the personality structure*.

**Hypothesis** **3** **(H3).** *The determinants of the support currently received are a patient’s positive orientation, openness, self-esteem, and lack of a sense of exclusion.*

**Hypothesis** **4** **(H4).** *The need for protective buffering support in the studied patients is influenced by a sense of alienation and exclusion because of the disease and the lack of openness to life experiences.*

**Hypothesis** **5** **(H5).** *The general sense of support during cancer treatment is influenced by patients’ attitude, personality structure, and general state of health.*

**Figure 1:**
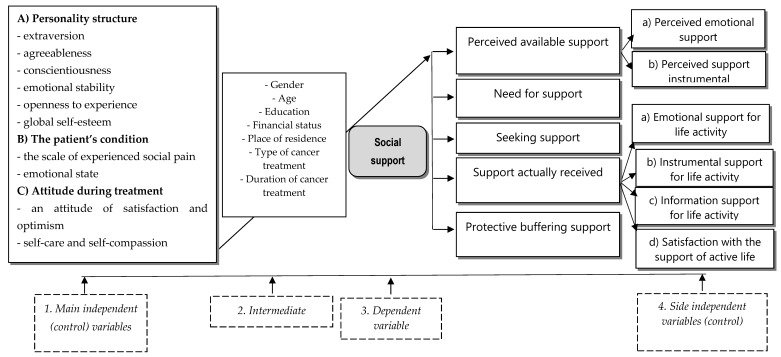
Theoretical model of social support for the studied patients during cancer treatment.

The assumptions of the study are shown in Figure 1, which graphically presents the theoretical model of the sense of social support in patients treated for cancer.

The theoretical model was developed based on literature on the subject [3,8,13,14,17,18,19,20], discussions with representatives of various specialists, and many years of our own experience in working with patients.

The model assumes that the sense of social support is influenced by the following main independent control variables: (a) personality structure, expressed through extraversion, agreeableness, conscientiousness, emotional stability, openness to experience, and global self-esteem; (b) a patient’s condition, expressed through a scale of experienced social pain and emotional state; and (c) attitude during the treatment process, expressed through satisfaction and optimism, as well as self-care and self-compassion.

The sense of social support depends on the intermediate variables of gender, age, education, financial status, place of residence, as well as the type and duration of cancer treatment.

The sense of social support is manifested by the side control variables of (a) the perceived available support, consisting of perceived emotional support and perceived instrumental support); (b) the need for support; (c) seeking support; (d) the support currently received, consisting of emotional support, instrumental support, information support, and satisfaction with the support received; and (e) protective buffering support (Figure 1).

In connection with the research problems undertaken, three groups of variables were distinguished: dependent variable, main independent (control) variables, and independent (control) side variables. The place of individual variables and their hypothetical dependence are illustrated in Figure 1 and Table 1.

The project was approved by the local Bioethics Committee of the Kraków Academy (Resolution No. 29/2015 of 14 May 2015). Participation in the research was voluntary. Having completed the questionnaire, each respondent put it into an unmarked envelope, ensuring anonymity.

### 2.2. Research Methods

The research project was cross-sectional and conducted using a diagnostic survey and questionnaire. To achieve the aim of the study, an original questionnaire was prepared to assess the sociodemographic and clinical situation of the respondents, and the other investigated variables were assessed with standardised questionnaires: the Berlin Social Support Scales, the Rosenberg Self-Esteem Scale, the Ten-Item Personality Inventory, the Positive Orientation Scale, the State Self-Compassion Scale, the Social Pain Thermometer, and the Emotion Thermometer.

The Berlin Social Support Scales (BSSS) determine the cognitive and behavioural dimensions of the social support experienced by the examined person. It consists of five scales: perceived support, the need for support, seeking support, the support currently received, and protective buffering support. This scale consists of eight items assessed on a 4-point Likert scale. The higher the score obtained by a respondent, the higher the level of perceived social support. The Cronbach’s α reliability index for this scale is 0.8617 [21].

The Rosenberg Self-Esteem Scale (SES) is a method for measuring global self-esteem, a construct that is regarded as a relatively stable trait, not a temporary state. Self-esteem is a positive or negative attitude towards oneself, a kind of global self-assessment. In this view, high self-esteem means the belief that one is a ‘good enough’ and worthwhile person. According to Rosenberg, low self-esteem means dissatisfaction with oneself, rejection of oneself. The Cronbach’s α coefficients for different age groups range from 0.81 to 0.83 [22].

The Ten-Item Personality Inventory (TIPI) is a short scale composed of ten items, each consisting of a pair of adjectives. They are selected in such a way as to reflect the possible and various characteristics falling within the scope of a given feature; to describe both the negative and positive poles of a given feature; not to be descriptions of the extreme intensity of both poles of the feature; not to contain negation; and to minimise redundancy in terms of feature descriptors. The Cronbach’s α reliability indices for the Polish version of the individual subscales are similar to the original indices [23].

The Positive Orientation Scale (POS) is a research tool that examines positive orientation, including self-esteem, life satisfaction, and optimism. The scale consists of eight items and the answers are given on a 5-point scale. The value of the Cronbach’s α coefficient ranges from 0.77 to 0.84, depending on the age of the respondents [24].

The State Self-Compassion Scale (SCSS) consists of 26 statements and measures five factors: self-kindness, self-judgement, common humanity, isolation, and mindfulness. Responses are given on a 5-point scale. The reliability of the scale was reported as 0.8521 [25].

The Social Pain Thermometer (SPT) examines three variables: injustice, exclusion and loss. Answers are given on a scale from 0 to 10. Each question is considered separately. It is a tool with confirmed parameters of psychometrics in health psychology and psycho-oncology.

The Emotion Thermometer (ET) is a tool that consists of five visual-analogue scales (such as levels on a thermometer) on which each respondent marks the level of emotions experienced in a specific situation. The respondents assessed their subjective feelings during treatment on the following scales: stress, anxiety, sadness, and the need for help. The higher the score on subsequent thermometers, the greater the intensity of a specific feeling. The ET consists of two factors: ET-1 (stress, anxiety, anger), Cronbach’s α–0.875, and ET-2 (depression, need for help), Cronbach’s α–0.789.

The study was conducted in the oncology wards (which are understood here as being radiotherapy and chemotherapy wards) of the leading cancer hospitals in Poland: Kraków, Lublin, Kielce, and Tarnów.

### 2.3. Participants

A total of 203 questionnaires were distributed, and 185 of them were returned. After an analysis of data completion, 170 were qualified for the study. The inclusion criteria for the study were: consent to participate, ongoing neoplastic process, radical cancer treatment (radiotherapy, chemotherapy, or combination therapy) in inpatient wards, as well as a mental state enabling understanding and independent completion of questionnaires.

The experimental group consisted of 170 patients treated for cancer. Over 70% were women. The mean age of the women was 55.73 years and the men 64.46 years, and these values were statistically significant (*p* < 0.0001). The result obtained indicates that the surveyed women were statistically younger than the surveyed men. This result is important for the assessment of the age of cancer incidence in the risk group (Table 2).

Most respondents were married or in an informal relationship (almost 75%) and declared completing secondary education (nearly 39%). Nearly 55% were retirees or disability pensioners, and slightly more than 31% worked full-time.

### 2.4. Statistical Analysis

The theoretical assumptions in the study and empirical research were conducted and verified according to the correlation-regression model [26]. In order to verify the theoretical model of the study more fully, the methods of structural equations and path analysis were used [27].

To create a sociodemographic and clinical characterisation of the studied groups, a statistical analysis of the size of the groups was carried out, including the percentage index for the total number of respondents. Due to the significant disproportion of the number of respondents in each group and a distribution of results that was inconsistent with the normal distribution, a comparative analysis of the groups of men and women for the ‘age’ variable was carried out using the nonparametric Mann–Whitney U test. The calculations were performed using the Statistica software package (Poland, Cracow).

Determinants of social support were searched based on multiple regression analysis, using the progressive stepwise method. An analysis of residual distribution was performed, and outliers (atypical observations) were removed from individual models (regression equations). The calculations were made using the Statistica software package.

In order to verify whether the theoretical model fit the data, structural equation modelling (SEM) was performed using AMOS software. The analysis of the results concerning the assumption of a multivariate normal distribution and outliers led to the selection of a group of 155 people for further analysis. The verification of the assumption of a multivariate normal distribution indicated that this assumption was not met in the analysed model, and so estimators resistant to breaking the assumption of a multivariate normal distribution were applied: MLR, MLM, and MLMV—the R package (environment) with the RStudio editor was used for this purpose.

## 3. Results

### 3.1. Clinical Characteristics of the Experimental Group

The vast majority of the people involved in the study (about 85%) had cancer for the first time or were in the first year of treatment (about 68%). Table 3 contains detailed data.

### 3.2. The Influence of Demographic and Clinical Variables on Social Support

The ‘gender’ variable differentiated the need for support between the studied women (M = 15.16) and men (M = 13.36) at a statistically significant level (*p* ≤ 0.01). There were no statistically significant differences in the remaining scales of support.

The ‘education’ variable did not affect the need for support in the studied patients.

The ‘place of residence’ variable did not show statistically significant differences, with the exception that respondents from large cities (M = 15.73) searched for support more than those from small towns (M = 14.09). Respondents from small towns (M = 3.76), on the other hand, had higher satisfaction with the support received (M = 3.57) than those living in large cities.

The ‘attitude to work’ variable (full-time work, disability pension, retirement pension) did not affect the need for support in the surveyed patients.

Patients who have previously undergone surgery sought support more intensely (M = 15.20) than those who had not had previous surgery (M = 13.97). The difference was statistically significant. Seeking support is associated with adopting a specific attitude that allows meeting the need for support.

On the other hand, respondents who had not had surgery showed a greater need for emotional support (M = 13.97) than those who had had previous surgery (M = 2.91), and the difference was statistically significant. Emotional support is treated as a kind of support—that is, the need for care from others in order to build a sense of security during the recovery process.

The number of previous surgeries statistically significantly differentiated patients on the scale of satisfaction with the support received. Patients after their second surgery were more satisfied (M = 3.67) with the support received than those after their first surgery (M = 3.88). There were no statistically significant differences in the remaining scales of support.

The ‘type of treatment’ variable differentiated patients at a statistically significant level (*p* ≤ 0.05) on the scale of the support currently received. The need for emotional support was lower for patients treated with chemotherapy than for those who had other types of treatment. There were no statistically significant differences in the remaining scales of support.

The duration of treatment also differentiated patients at a statistically significant level in the scale of the perceived information support. This concerns the relationship between patients treated for 1–3 months (M = 13.40) and for 4–12 months (M = 14.80, *p* ≤ 0.01); for 1–3 months (M = 13.40) and 1–2 years (M = 14.34; *p* ≤ 0.05); and for 4–12 months (M = 14.80) and 2–5 years (M = 13.91; *p* ≤ 0.05).

In the scale of the need for support, statistically significant correlations occurred in patients treated for 1–3 months (M = 11.79) and 2–5 years (M = 10.74; *p* ≤ 0.05); for 4–12 months (M = 12.55) and 2–5 years (M = 10.74; *p* ≤ 0.001); and for 1–2 years (M = 12.59) and 2–5 years (10.74; *p* ≤ 0.01).

In the scale of seeking support, statistically significant correlations occurred in patients treated for 1–3 months (M = 14.12) and 4–12 months (M = 15.42; *p* ≤ 0.05); for 4–12 months (M = 15.42) and 2–5 years (M = 13.26; *p* ≤ 0.05); and for 1–2 years (M = 15.38) and 2–5 years (M = 13.26; *p* ≤ 0.05).

In the scale of satisfaction with the support received, there was a significant correlation between people treated for 4–12 months (M = 3.82) and 2–5 years (M = 3.84; *p* ≤ 0.05).

The ‘duration of treatment’ variable in all the scales discussed above differentiated patients treated for 4–12 months, whose sense of support was higher than that of patients treated for 2–5 years.

Our study showed that the longer a patient was treated, the lower their sense of support was.

### 3.3. Analysis of Determinants of Social Support

The data obtained in our study were subjected to statistical analysis, and correlations with individual components of social support were calculated. The variables that were statistically significant in explaining the variables of the BSSS questionnaire subscales or showed high dependence coefficients are presented below.

The study showed that perceived emotional support was conditioned by the variables of the POS (β = 0.30) and the ‘exclusion variable’ (β = −0.19) on the SPT.2 scale (Table 4). They were important for explaining the ‘perceived emotional support’ variable. The positive correlation obtained between the POS and perceived emotional support indicates that people who had a positive view about themselves and the world, and experienced life satisfaction despite various difficult situations, noticed and perceived the provided emotional support to a greater extent. On the other hand, people with a tendency to a negative assessment of a situation and themselves, who had no prospects for the future, would barely be aware of emotional support from relatives or medics.

Regression analysis showed that people with no sense of social exclusion were open-minded and perceived the emotional support they received from others.

The anger scale (β = −0.12) with a negative correlation was qualified to explain the variable of perceived emotional support in the studied patients, but it was not statistically significant.

The analysis of the correlation coefficient showed that the variables were correlated at a medium level (R = 0.46). The variables of the POS and exclusion (SPT.2) indicate that perceived emotional support was an important area of social support, explaining 21% of the variance (R^2^ = 0.21). The remaining percentage was due to the influence of independent variables that were not part of the regression equation.

Perceived instrumental support was conditioned by the POS (β = 0.38). The positive correlation obtained between the scales indicates that patients who were characterised by positive self-esteem and attached importance to the positive aspects of life despite various difficult situations would perceive and appreciate more the instrumental support provided to them in the form of concrete help. On the other hand, patients who were prone to a negative assessment of a situation or themselves had difficulty noticing instrumental support given to them. The exclusion (β = −0.14) and anger (β = −0.10) scales were additionally included in the explanation of the ‘perceived instrumental support’ variable in the studied patients, showing a negative correlation, which was, however, not statistically significant.

The analysis of the correlation coefficient shows that the analysed variables were related to each other at a medium level (R = 0.49). The ‘POS’ variable indicates that perceived instrumental support was an important area of social support, explaining 24% of the variance (R^2^ = 0.24). The remaining percentage was due to the influence of independent variables that were not part of the regression equation.

Our study showed that the need for support was statistically significantly conditioned by the need for help (β = 0.28) and anger (β = −0.29). The studied patients treated for cancer who demonstrated the need for help were characterised by an increased need for support, unlike the patients who did not show such a need. The negative correlation between the variables (β = −0.29) indicates that patients who did not experience negative emotions, such as anger, could be characterised by an increased need for support during cancer treatment. Experiencing negative emotions blocked both the need for and openness to support (Table 5).

The scales of loss (β = 0.15) and self-esteem (β = 0.11) were included in the explanation of the ‘need for support’ variable in the studied patients, showing a positive correlation, which was, however, not statistically significant.

The analysis of the correlation coefficient shows that the analysed variables were related to each other at a medium level (R = 0.33). The ‘need for help’ and ‘anger’ variables indicate that the need for support was an important area of social support, explaining 11% of the variance (R^2^ = 0.11). The remaining percentage was due to the influence of independent variables that were not part of the regression equation.

The results in Table 6 show that seeking support was conditioned by the need for help (β = 0.43), anger (β = −0.27) and extroversion (β = 0.19). The surveyed patients who signalled the need for help could be characterised by a higher commitment to seeking support than those who said that they did not feel such a need (Table 6).

People who did not experience anger and irritation sought support more intensely during treatment than those experiencing negative emotions during this period (a negative correlation between the studied variables).

The positive relationship between extraversion and seeking support indicates that patients characterised by the ease of expressing thoughts, emotions or feelings, openness in establishing interpersonal contacts, and a strong need for action were open to and looked for support during treatment. On the other hand, patients prone to shyness, uncertainty, and withdrawal in interpersonal relations found it difficult to actively seek support during the treatment process.

To explain the ‘seeking support’ variable, openness to experience, emotional stability (showing a negative correlation), and self-compassion (showing a positive correlation) were additionally qualified. However, they were not statistically significant in the studied group.

The analysis of the correlation coefficient shows that the variables need for help, anger, and extraversion explained seeking support and were interrelated at a medium level (R = 0.40), explaining 16% of the variance (R^2^ = 0.16). The remaining percentage was due to the influence of independent variables that were not part of the regression equation (Table 6).

The next stage of the study involved analysis of the support currently received, which consisted of the following components: emotional, instrumental and information support, and satisfaction with the support received. The results are summarised in Table 7, Table 8, Table 9, Table 10 and Table 11, followed by detailed analysis and interpretation.

The support currently received was conditioned by the POS (β = 0.22) and exclusion obtained from the SPT.2 questionnaire (β = −0.19) (Table 7). A positive correlation with the POS means that the surveyed patients who experienced life satisfaction during treatment and had positive self-esteem more noticed the support currently provided in difficult situations. Patients who showed a negative assessment of the situation or themselves had difficulty noticing the support currently received. Respondents with no sense of social exclusion were able to perceive the support currently received.

The self-esteem scale, which showed a positive relationship, was additionally qualified to explain the variable of support currently received by the group of patients but was not statistically significant.

The analysis of the correlation coefficient shows that the analysed variables were correlated at a medium level (R = 0.39), explaining 15% of the variance (R^2^ = 0.15). The remaining percentage was due to the influence of independent variables that were not part of the regression equation (Table 7).

Our study shows that emotional support was conditioned by the SES variables (β = 0.16). The positive correlation obtained shows that people characterised by a positive and high assessment of themselves and their actions were open to emotional support from other people. Patients who evaluated themselves negatively were unable to accept emotional support (Table 8).

In explaining the ‘emotional support’ variable, the ‘exclusion’ scale (SPT.2) was additionally distinguished, showing a negative relationship, but it was not statistically significant in the studied group.

The analysis of the correlation coefficient shows that the analysed variables were related to each other at a medium level (R = 0.24). SES variables indicated that emotional support was an important area of social support, explaining 6% of the variance (R^2^ = 0.06). The remaining percentage of variability was due to the influence of independent variables that were not part of the regression equation.

The study shows that exclusion (β = −0.29) and the POS (β = 0.16) determined instrumental support. Patients characterised by no sense of social exclusion and who were actively participating in activities and social life perceived the instrumental support provided to them. In turn, patients who felt withdrawn and alienated from social activities found it difficult to accept instrumental support during cancer treatment. Those characterised by high satisfaction with life and positive self-esteem during treatment were able to benefit from instrumental support. Patients who showed a negative assessment of the situation or themselves found it difficult to accept instrumental support (Table 9).

The ‘agreeableness’ scale (β = 0.12), showing a positive correlation, qualified for the explanation of instrumental support, but was not statistically significant in the studied group.

The analysis of the correlation coefficient shows that the analysed variables were correlated at a medium level (R = 0.40) and that instrumental support was an important area of social support, explaining 16% of the variance (R^2^ = 0.16). The remaining percentage was due to the influence of independent variables that were not part of the regression equation.

The study shows that information support was conditioned by the POS (β = 0.30). The positive correlation between the scales indicates that patients characterised by life satisfaction, attaching importance to the positive aspects of life, and having positive self-esteem during the treatment process were able to accept information support. Patients who did not attach importance to the positive aspects of life or did not notice them found it difficult to accept information support (Table 10).

The explanation of the ‘information support’ variable was additionally supplemented by the following scales: anger (β = −0.10), showing a negative relationship; SES (β = 0.12), showing a positive correlation; anxiety (β = −0.10), showing a negative relationship; and stress (β = 0.26), showing a positive relationship. They were not statistically significant in the studied group of patients.

The analysis of the correlation coefficient shows that the analysed variables were correlated with each other at a medium level (R = 0.42) and explained 18% of the variance (R^2^ = 0.18). The remaining percentage of variability was due to the influence of independent variables that were not part of the regression equation (Table 10).

The results in Table 11 show that satisfaction with the support received was determined by the exclusion scale (SPT.2) (β = −0.28). The negative correlation obtained indicates that patients who, despite their disease, actively participated in activities and social life and could be characterised by no sense of social exclusion seen and were able to appreciate and be satisfied with support in the treatment process. Patients who felt withdrawn and alienated from social activities during the treatment process found it difficult to be satisfied with and show gratitude for the support provided to them.

The POS (β = 0.10), showing a positive relationship, was additionally included in the explanation of the ‘satisfaction with the support received’ variable, but was not statistically significant (Table 11).

The analysis of the correlation coefficient shows that the analysed variables correlated with each other at a medium level (R = 0.32). The ‘exclusion’ variable (SPT.2) indicates that satisfaction with the support received was an important area of social support, explaining 10% of the variance (R^2^ = 0.10). The remaining percentage of variability was due to the influence of independent variables that were not part of the regression equation.

Our study shows that protective buffering support was conditioned by exclusion (SPT.2) (β = 0.16) and openness to experience (O) (β = −0.16).

A positive correlation with exclusion means that patients who were characterised by no involvement and activity in life did not join in activities and social life and experienced a sense of social exclusion and showed a special need for protective buffering support. This type of support was not needed by patients who were actively involved in their individual and social lives and did not feel alienated or excluded from society.

On the other hand, patients who were not open to experiences and accepted difficult situations in life with fear and uncertainty showed the need for protective buffering support. In turn, patients who were open-minded and coped with new life situations during cancer treatment did not need such support.

The SCSS (β = −0.13), showing a negative correlation, was additionally used to explain the ‘protective buffering support’ variable in the group of patients, but it was not statistically significant.

The analysis of the correlation coefficient shows that the analysed variables were correlated at a medium level (R = 0.32) and that protective buffering support was an important area of social support, explaining 10% of the variance (R^2^ = 0.10). The remaining percentage of variability was due to the influence of independent variables that were not part of the regression equation (Table 12).

### 3.4. Verification of the Research Model

In order to verify the theoretical model, structural equations were modelled. Initial data analysis indicated the necessity to remove outliers. A sample of *n* = 155 was selected, which allowed satisfactory indicators of goodness-of-fit for the model to be obtained. Due to the failure to meet the assumption of a multivariate normal distribution, robust estimators were used in the calculations, which also confirmed the fit of the model to the data (insignificant levels of significance).

The statistical parameters obtained indicate that the model explained well the previously assumed hypothetical theoretical model in the group of patients undergoing cancer treatment: Chi-squared = 16.333 was statistically insignificant, RMSEA = 0.034 was good, CFI = 0.980, GFI = 0.901 were greater than 0.901 and approached 1, the RMSEA = 0.034, and the upper confidence interval was 0.053. This means that the model was a good fit (Table 13).

## 4. Discussion

Based on the study and statistical analysis, a well-fitting verified research model was obtained. It applies to cancer patients treated for cancer (radio- or chemotherapy or combination therapy) in inpatient wards.

In the group of examined patients, personality structure expressed by extraversion (E) (I–E) (0.65), agreeableness (A) (0.44), conscientiousness (C) (0.43), emotional stability (ES) (0.42), openness to experience (O) (0.36) and self-esteem (SES) (0.66) influenced the support experienced during treatment (0.40) due to correlations with their health condition during cancer treatment (0.40) and their attitude developed to the disease (0.64).

The above correlations of variables made it possible to conclude that the level of support experienced by the studied patients is influenced by such personality traits as openness and optimistic thinking; personal trust of and a sincere attitude towards another person; perseverance in pursuing goals; internal conviction about the rightness of actions; emotional stability and maturity; openness to life experiences; and the ability to positively re-evaluate difficult situations, as well as adequate and realistic self-assessment of one’s abilities and resourcefulness.

The study showed that in the personality structure of patients, extraversion (E) was additionally associated with emotional stability (ES). The result obtained (0.27) indicates that such features as the need for relationships with others, social activity, sociability and friendliness were related to emotional stability and control. These are the features of a mature personality structure, which is a very important modifier in experiencing difficult life situations. Agreeableness was related to conscientiousness at the level of 0.27, which means that, in the personality structure of the studied patients, there was a significant correlation between the features of sympathy, altruism, and helping others and the features of persistence and motivation in achieving goals and the inner conviction of the rightness of actions. Moreover, conscientiousness was additionally associated with emotional stability, where the relationship was negative and equalled −0.19, and emotional stability was correlated with openness to experience at the level of −0.29, meaning it was also negative. These correlations indicate that in the personality structure of the studied patients, an insufficient level of conscientiousness and perseverance negatively affected emotional stability, introducing emotional anxiety and tension. Moreover, the lack of emotional stability blocked openness to experience, which in turn may decrease the ability to re-evaluate the difficult situations experienced. In the case of oncological treatment, such features can negatively affect the entire therapy process.

The general condition of patients treated for cancer, as reflected by the social pain thermometer (SPT) (0.67), the level of stress (0.80), anxiety (0.82), depressive states (0.81), anger (0.67), and the need for help (0.76) influenced support during treatment (0.40) through correlations with the personality structure (0.40) and the patient’s attitude (−0.52). The results obtained and the positive correlation between the variables indicate that the overall positively assessed health condition of the surveyed patients in the treatment process was influenced by the control of the sense of injustice, no sense of social isolation, no sense of loss, control of the levels of stress and internal anxiety experienced, no depression or low mood, control of negative emotions such as anger, and the need for and openness to accepting help from others.

The health condition experienced influenced the attitude of cancer patients (−0.52). The negative correlation indicates that those who perceived the disease as an injustice and loss were unable to cope with stress and anxiety and experienced depression and anger in the event of cancer, thus shaping a negative attitude towards the entire treatment process. The variable personality structure manifested by the above-described scales influenced the attitude that patients adopted during cancer treatment (0.64). The correlation was positive and the index obtained indicates a high correlation between the scales. The patient’s attitude was also influenced by the ‘patient’s health’ variable, which was manifested in the above-mentioned scales. The correlation level was −0.52 and was statistically high. The attitude of patients undergoing cancer treatment, shaped by the variables of personality structure (0.64), health condition (−0.52), and their components influenced the level of support perceived by the studied patients in the treatment process. The correlation obtained was 0.40 and was assessed at a medium level.

The main studied variable was ‘support’, which in patients during cancer treatment manifested itself in perceived emotional support (PES, 0.92), perceived instrumental support (PIS, 0.86), and support currently received (SCR) (0.59). The examined support was additionally associated with complementary support (CS2) at the level of 0.18 in the studied patients, manifested primarily in the need for support (NS) (0.65) and the sense of support (SP) (0.83). The correlation was positive.

The dependence obtained and the correlation between the studied variables indicate that the support that patients received during cancer treatment was expressed, in particular, through its emotional and instrumental form and the level of support currently received.

The index of the explained variable, which was support in the group of cancer patients, was at the level of 0.40, which means that the remaining percentage of the explanatory variables was not included in the study and requires further research.

In response to the formulated research hypotheses, it can be stated that all were positively verified. The most important conclusions are summarised below.

**Figure 2:**
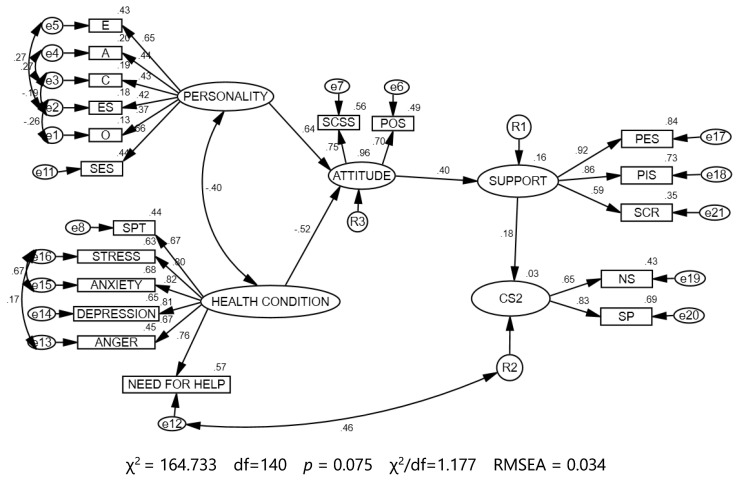
Model of social support for cancer patients during cancer treatment. E—Extraversion, A—Agreeableness, C—conscientiousness, ES—Emotional Stability, O—Openness to Experience, SES—Rosenberg Self-Esteem Scale, SPT—Social Pain Thermometer, SCSS—State Self-Compassion Scale, POS—Positive Orientation Scale, PES—Perceived Emotional Support, PIS—Perceived Instrumental Support, SCR—Support Currently Received, NS—Need for Support, SP—Sense of Support, CS2—Complementary Support.

The surveyed patients, who were characterised by positive self-esteem and experience of life satisfaction despite various difficult situations, perceived the emotional and instrumental support given to them to a greater degree. Hypothesis 1 was positively verified. The study and the regression analysis showed that the positive relationship between the POS and PES scales indicated that, despite various difficult situations, patients who experienced life satisfaction and had a positive attitude noticed and perceived the emotional and instrumental support provided to them more intensely. The lack of such an attitude and the accompanying negative emotional states made it difficult to perceive emotional and instrumental support. The path analysis confirmed the relationship included in the hypothesis. The statistical analysis showed that the POS was related to the attitude of patients during treatment and that in this configuration it influenced the perceived support (Figure 2).

During cancer treatment, patients who showed their need for help and did not experience negative emotions were characterised by an increased need for support. Hypothesis 2 was positively confirmed. The study and the regression analysis confirmed that the hypothesis was correct. The cancer patients who showed the need for help and did not experience negative emotions during treatment were characterised by an increased need and search for support. The path analysis verifying the theoretical research assumptions confirmed the above dependence. The features of openness of the studied patients were within the personality structure, which influenced support through a shaped attitude during the treatment process. The need for help and negative emotions experienced during cancer treatment were related to the ‘patient’s health’ variable. Personality traits and health condition were interrelated and influenced the need and search for support through changing attitudes during the treatment process.

Frambes et al. noted that the lack of identification of support may contribute to the occurrence of anxiety and depression [9]. Langbecker found that addressing barriers to seeking help can improve the bio-psychosocial well-being of patients [28].

Patients who attached importance to the positive aspects of life and experienced no sense of social alienation during treatment noticed the instrumental and information support currently received and felt satisfied with this support. Perceived emotional support was influenced by positive self-esteem. The hypothesis was positively verified. The multiple regression analysis showed that patients who experienced life satisfaction and had a favourable self-esteem during the treatment process were able to perceive and appreciate the support given to them.

The path analysis verifying the theoretical research assumptions also confirmed that the hypothesis was correct. The features of self-esteem and openness in the personality structure were associated with patients’ attitude, which was manifested in positive orientation, among others. The attitude of patients during treatment was influenced by their health condition, the assessment of which was also affected by the lack of a sense of exclusion. This set of variables allowed patients to notice, appreciate, and take advantage of the support provided to them.

If patients experienced a sense of social exclusion during cancer treatment and were not open to experiences, they reacted with anxiety and uncertainty and showed a special need for protective buffering support. The hypothesis was confirmed. The multiple regression analysis showed that features such as no involvement and openness, as well as low activity in life and a sense of social exclusion, increased the need for buffering protective support. The pathway analysis showed that the variable of openness was within the personality structure, which was related to the attitude adopted by patients during the treatment process. In turn, a sense of exclusion was one of the variables related to health condition which also influenced the attitude of patients during treatment. This configuration of variables influenced the experience of a general sense of support. The pathway analysis did not distinguish buffering protective support as a characteristic variable through which the overall support was manifested in the studied patients. This can be explained by the fact that not many of the studied patients had highly saturated features, such as a sense of alienation and the lack of openness. However, due to the results of the multiple regression analysis, in future studies it would be worth paying attention to patients experiencing such negative conditions during cancer.

The sense of support in cancer treatment was influenced by the attitude adopted by patients during treatment, their personality structure, and general health condition.

The hypothesis was positively verified, as evidenced by the path analysis model. The level of support experienced by the surveyed patients was influenced by their attitude, which was conditioned by such personality traits as openness, optimism, attitude towards others, perseverance in pursuing goals, emotional stability, and maturity and by health condition. The subjectively assessed health condition was influenced by the sense of control of experienced stress and anxiety, no sense of social isolation and loss, and control of negative emotions, as well as by the need for and openness to help from others. This shows the importance of these variables in the further analysis of the level and type of support provided to patients during cancer treatment.

### Study Limitations

Our study was cross-sectional. A longitudinal study would allow the dynamics of changes in the perception of social support to be traced.

Increasing the number of research participants, including a comparable number of men and women in the study, would make it possible to check how the tested model of social support works in a wider research group. Taking account of, for example, existential aspects in experiencing cancer would direct the research towards a comparative assessment of the perception of multidimensional support experienced by patients.

The level of explained variance of the individual variables included in the research model indicates that the sense of support, in addition to the discussed variables, also depended on other factors not included in the study. Therefore, it is worth continuing the search for predictors of social support in oncological patients and extending the research aspect to include caregivers’ experience of support, as well as extending the research to include a multidimensional analysis of the specificity of support in the patient–caregiver dyad model.

Our study has both a cognitive and practical value and broadens the knowledge in the field of helping patients to survive the disease and cope with the recovery process. The research results and conclusions can be used to develop multidimensional forms of support for cancer patients and their relatives.

## 5. Conclusions

The research material obtained and its analysis constitute a unique contribution to the development of a support system for patients during cancer treatment.

Patients who have had previous surgery, have a greater need for support.

Emotional support is needed more by patients who have not had any surgery or related experience.

The longer a patient is treated for cancer, the lower their sense of information support, satisfaction with the support received, need for support, and seeking of support.

The research results can be introduced into evidence-based medical practice to significantly improve the quality of nursing and medical care. The social support system, its type and scope, vary depending not only on the stage of treatment but also on personality structure. The surveyed patients who were characterised by positive self-esteem and experience of life satisfaction despite various difficult situations perceived more and more intensely the emotional and instrumental support given to them. During cancer treatment, patients who showed the need for help and did not experience negative emotions were characterised by an increased need for support.

## Figures and Tables

**Table 1:** Investigated variables and applied research tools.

**Main Variable**	**Index**	**Research Method**
Social support	Perceived available support The need for support Seeking support Currently received support Protective buffering support	BSSS—Berlin Social Support Scales
**Independent (Control) Side Variables**	**Index**	**Research Method**
Types of support	- Perceived emotional support - Perceived instrumental support - Emotional support - Instrumental support - Information support - Satisfaction with support	BSSS—Berlin Social Support Scales
**Main Independent** **(Control) Variables**	**Index**	**Research Method**
1. Personality structure	- Extroversion - Agreeableness - Conscientiousness - Emotional Stability - Openness to Experience - Global Self-Esteem	(1) TIPI—Ten-Item Personality Inventory (2) SES—Rosenberg Self-Esteem Scale
2. The patient’s condition	- The scale of experienced social pain - Emotional state	(1) SPT—Social Pain Thermometer (2) ET—Emotion Thermometer
3. Attitude during the treatment process	- Attitude of satisfaction and optimism - Self-care and self-compassion	(1) POS—Positive Orientation Scale (2) SCSS—State Self-Compassion Scale
**Intermediate Variables**	**Index**	**Method**
	- Gender - Age - Education - Financial status - Place of residence - Type of cancer treatment - Duration of cancer treatment	Personal questionnaire

**Table 2:** Sociodemographic characteristics of the experimental group.

Variables	*n* Respondents (*n* = 170)	% Respondents
Gender		
Female	120	70.59
Male	50	29.41
Age		
19–30	7	4.12
31–39	13	7.65
40–49	24	14.12
50–59	38	22.35
60–69	50	29.41
70–79	30	17.65
80–89	8	4.71
Marital status		
Married	119	70.00
Single	13	7.65
Widowed	27	15.88
Informal relationship	8	4.71
Other	3	1.76
Education		
Primary	9	5.29
Vocational	54	31.76
Secondary	66	38.82
Higher	41	24.12
Financial status		
Full-time job	53	31.18
Part-time job or a civil law contract	10	5.88
Disability pension	35	20.59
Retirement pension	57	33.53
Benefits	5	20.59
Does not work	10	24.12
Place of residence		
City	53	31.18
Town	61	35.88
Village	56	32.94

**Table 3:** Clinical characteristics of the experimental group.

Variables	*n* Respondents (*n* = 170)	% Respondents
Current treatment		
Combination therapy	75	44.11
Chemotherapy	57	33.52
Radiotherapy	45	22.37
Treatment period (*n* = 164)		
1–3 months	57	34.76
4–12 months	55	33.54
1–2 years	29	17.68
2–5 years	23	14.02
First incidence of cancer		
yes	145	85.29
no	25	14.71
Earlier surgery for cancer		
yes	91	53.53
no	79	46.47

**Table 4:** Determinants of variables for perceived support.

Variables	BETA	Stat. Error BETA	B	Stat. Error B	*t*	*p*
Perceived emotional support						
Constant term			11.61	0.92	12.57	0.0001
POS	0.30	0.07	0.11	0.03	3.96	0.0001 ***
SPT.2-Exclusion	−0.19	0.08	−0.13	0.05	−2.38	0.019 *
ANGER	−0.12	0.08	−0.07	0.05	−1.46	0.147
R = 0.46, R^2^ = 0.21, F_(3.163)_ = 14.52, *p* < 0.001						
Perceived instrumental support						
Constant term			10.27	1.00	10.32	0.0001
POS	0.38	0.07	0.16	0.03	5.23	0.0001 ***
SPT.2-Exclusion	−0.14	0.08	−0.11	0.06	−1.88	0.062
ANGER	−0.10	0.08	−0.07	0.05	−1.25	0.212
R = 0.49, R^2^ = 0.24, F_(3.164)_ = 17.77, *p* < 0.001						

R—Multiple correlation coefficient; *** *p* < 0.0001, * *p* < 0.05, R^2^—Multiple determination coefficient; F, *p*—the significance of the equation; *t*—Student’s *t*-test.

**Table 5:** Determinants of variables for the need for support.

Variables	BETA	Stat. Error BETA	B	Stat. Error B	*t*	*p*
Constant term			9.47	1.43	6.63	0.0001
Need for support	0.28	0.09	0.19	0.06	3.10	0.002 **
Anger	−0.29	0.09	−0.19	0.06	−3.28	0.001 ***
SPT.3-Loss	0.15	0.09	0.11	0.06	1.78	0.078
SES	0.11	0.08	0.06	0.04	1.43	0.155

R = 0.33, R^2^ = 0.11, F(4.165) = 5.04, ** *p* < 0.001, *** *p* < 0.0001, R—Multiple correlation coefficient; R^2^—Multiple determination coefficient; F, *p*—the significance of the equation; *t*—Student’s *t*-test.

**Table 6:** Determinants of the ‘seeking support’ variable (SS).

Variables	BETA	Stat. Error BETA	B	Stat. Error B	*t*	*p*
Constant term			12.24	2.20	5.57	0.0001
Need for help	0.43	0.09	0.48	0.10	4.95	0.0001 ***
Anger	−0.27	0.09	−0.29	0.10	−3.05	0.003 **
E—Extroversion	0.19	0.09	0.44	0.20	2.16	0.032 *
O—Openness to experience	−0.14	0.08	−0.41	0.24	−1.70	0.092
SE—Stable emotions	−0.12	0.09	−0.28	0.20	−1.43	0.156
SCSS	0.10	0.09	0.06	0.05	1.08	0.283

R = 0.40, R^2^ = 0.16, F_(6.163)_ = 5.26, *** *p* < 0.0001, ** *p* < 0.001, * *p* < 0.05, R—Multiple correlation coefficient; R^2^—Multiple determination coefficient; F, *p*—the significance of the equation; *t*—Student’s *t*-test.

**Table 7:** Determinants of the ‘support currently received’ variable (SCR).

Variables	BETA	Stat. Error BETA	B	Stat. Error B	*t*	*p*
Constant term			2.61	0.23	11.39	0.0001
POS	0.22	0.08	0.01	0.01	2.61	0.010 **
SPT.2-Exclusion	−0.19	0.08	−0.02	0.01	−2.46	0.015 *
SES	0.10	0.08	0.01	0.01	1.17	0.245

R = 0.39, R^2^ = 0.15, F_(3.164)_ = 9.74, ** *p* < 0.001, * *p* < 0.05, R—Multiple correlation coefficient; R^2^—Multiple determination coefficient; F, *p*—the significance of the equation; *t*—Student’s *t*-test.

**Table 8:** Determinants of the ‘emotional support’ variable (ES).

Variables	BETA	Stat. Error BETA	B	Stat. Error B	*t*	*p*
Constant term			2.60	0.21	12.44	0.0001
SES	0.16	0.08	0.01	0.01	2.05	0.042 *
SPT.2-Exclusion	−0.13	0.08	−0.01	0.01	−1.67	0.096

R = 0.24, R^2^ = 0.06, F_(2,167)_ = 5.03, * *p* < 0.05, R—Multiple correlation coefficient; R^2^—Multiple determination coefficient; F, *p*—the significance of the equation; *t*—Student’s *t*-test.

**Table 9:** Determinants of the ‘instrumental support’ variable.

Variables	BETA	Stat. Error BETA	B	Stat. Error B	*t*	*p*
Constant term			3.05	0.25	12.10	0.0001
SPT.2-Exclusion	−0.29	0.08	−0.05	0.01	−3.79	0.0001 ***
POS	0.16	0.08	0.02	0.01	1.98	0.050 *
U—Agreeableness	0.12	0.08	0.05	0.03	1.54	0.125

R = 0.40, R^2^ = 0.16, F_(3.164)_ = 10.54, *** *p* < 0.0001, * *p* < 0.05, R—Multiple correlation coefficient; R^2^—Multiple determination coefficient; F, *p*—the significance of the equation; *t*—Student’s *t*-test.

**Table 10:** Determinants of the ‘information support’ variable.

Variables	BETA	Stat. Error BETA	B	Stat. Error B	*t*	*p*
Constant term			1.97	0.43	4.56	0.0001
POS	0.30	0.08	0.04	0.01	3.59	0.0001 ***
ANGER	−0.10	0.09	−0.02	0.02	−1.05	0.294
SES	0.12	0.08	0.02	0.01	1.48	0.141
ANXIETY	−0.26	0.15	−0.06	0.03	−1.81	0.073
STRESS	0.26	0.16	0.05	0.03	1.66	0.099

R = 0.42, R^2^ = 0.18, F_(5.162)_ = 7.07, *** *p* < 0.0001, R—Multiple correlation coefficient; R^2^—Multiple determination coefficient; F, *p*—the significance of the equation; *t*—Student’s *t*-test.

**Table 11:** Determinants of the ‘satisfaction with support received’ variable (SSR).

Variables	BETA	Stat. Error BETA	B	Stat. Error B	*t*	*p*
Constant term			3.62	0.22	16.14	0.0001
SPT.2-Exclusion	−0.28	0.08	−0.05	0.01	−3.65	0.0001 ***
POS	0.10	0.08	0.01	0.01	1.25	0.214

R = 0.32, R^2^ = 0.10, F_(2.165)_ = 9.66, *** *p* < 0.000, R—Multiple correlation coefficient; R^2^—Multiple determination coefficient; F, *p*—the significance of the equation; *t*—Student’s *t*-test.

**Table 12:** Determinants of the ‘protective buffering’ variable.

Variables	BETA	Stat. Error BETA	B	Stat. Error B	*t*	*p*
Constant term			3.70	0.44	8.40	0.0001
SPT.2-Exclusion	0.16	0.08	0.04	0.02	1.97	0.050 *
O—Openness to experience	−0.16	0.08	−0.11	0.05	−2.18	0.030 *
SCSS	−0.13	0.08	−0.02	0.01	−1.48	0.141

R = 0.32, R^2^ = 0.10, F_(3.166)_ = 6.39, * *p* < 0.05, R—Multiple correlation coefficient; R^2^—Multiple determination coefficient; F, *p*—the significance of the equation; *t*—Student’s *t*-test.

**Table 13:** Goodness-of-fit indices for the research model of social support.

Goodness-of-Fit Indices		Value
**Tucker Lewis Index**	TLI	0.975
**Comparative Fit Index**	CFI	0.980
**Relative Fit Index**	RFI	0.856
**Normed Fit Index**	NFI	0.882
**Critical N**	CN	158
**Standardised RMR**	SRMR	0.061
**Root-Mean-Square Error of Approx. (conf. interval 90%)**	RMSEA (LO-HI90)	0.034 (0.0001–0.053)
**p of Close Fit**	PCLOSE	0.910
**Goodness-of-Fit Index**	GFI	0.901
**Adjusted Goodness-of-Fit Index**	AGFI	0.865
**Default model**	AIC	264.733
**Saturated model**	AIC	380.0001
**Independence model**	AIC	1433.112

## Data Availability

The data presented in this study are available on request from the corresponding author.
